# COVID‐19 in two infants in China

**DOI:** 10.1002/iid3.319

**Published:** 2020-07-02

**Authors:** Anni Li, Xiaolin Zhou, Wei Lu, Yuanhong Zhou, Qiang Liu

**Affiliations:** ^1^ Yichang Central People's Hospital, The First College of Clinical Medical Science China Three Gorges University Yichang China

**Keywords:** COVID‐19, infant, infection, SARS‐CoV‐2

## Abstract

**Introduction:**

Since December 2019, severe acute respiratory syndrome coronavirus 2 (SARS‐CoV‐2) has spread rapidly around the world and caused more than 487 000 infections and 22 000 deaths worldwide.

**Methods:**

We report two infant cases with coronavirus disease 2019 (COVID‐19) in Yichang, Hubei, China. The younger of the two is only 5‐months old. We recorded their clinical manifestations, epidemiological history, laboratory examination, and treatment in detail. In addition, we provide computed tomographic images of their chest, which are the most serious imaging manifestation among the infants recorded so far.

**Results:**

Although both of them eventually recovered and were discharged from the hospital, they were complicated with varying degrees of liver and myocardial injury. In addition, one of them was complicated with mycoplasma pneumoniae infection.

**Conclusions:**

Pediatricians should consider the potential risks of developing severe illness of infants infected by SARS‐CoV‐2 and take them seriously.

## Graphical abstract

The report describes the characteristics of the disease in two infants caused by severe acute respiratory syndrome coronavirus 2 (SARS‐CoV‐2). Compared with adult infected patients, most of the affected children were milder. However, it should be noted that severe infection is not rare, so clinicians should pay attention to the diagnosis and treatment of children with coronavirus disease 2019 (COVID‐19).

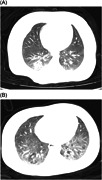

## 1 INTRODUCTION

The severe acute respiratory syndrome coronavirus 2 (SARS‐CoV‐2) is a β coronavirus with envelope and 60 to 140 nm in diameter, which is a global urgent public health concern because of its alarmingly high infection rates. SARS‐CoV‐2 contributes to the decrease of lymphocytes, the increase of the level of C‐reactive protein, and the secretion of a large number of inflammatory factors causing cytokine storm. It can lead to multiple organ failure in severe cases.^1^ However, there is no specific treatment in clinic. The previous view suggests that infants are not at a higher risk for severe coronavirus disease 2019 (COVID‐19) pneumonia.^2^, ^3^, ^4^ We herein report two infant cases with COVID‐19 in Yichang, Hubei, China.

## 2 CASE PRESENTATION

**Figure 1:**
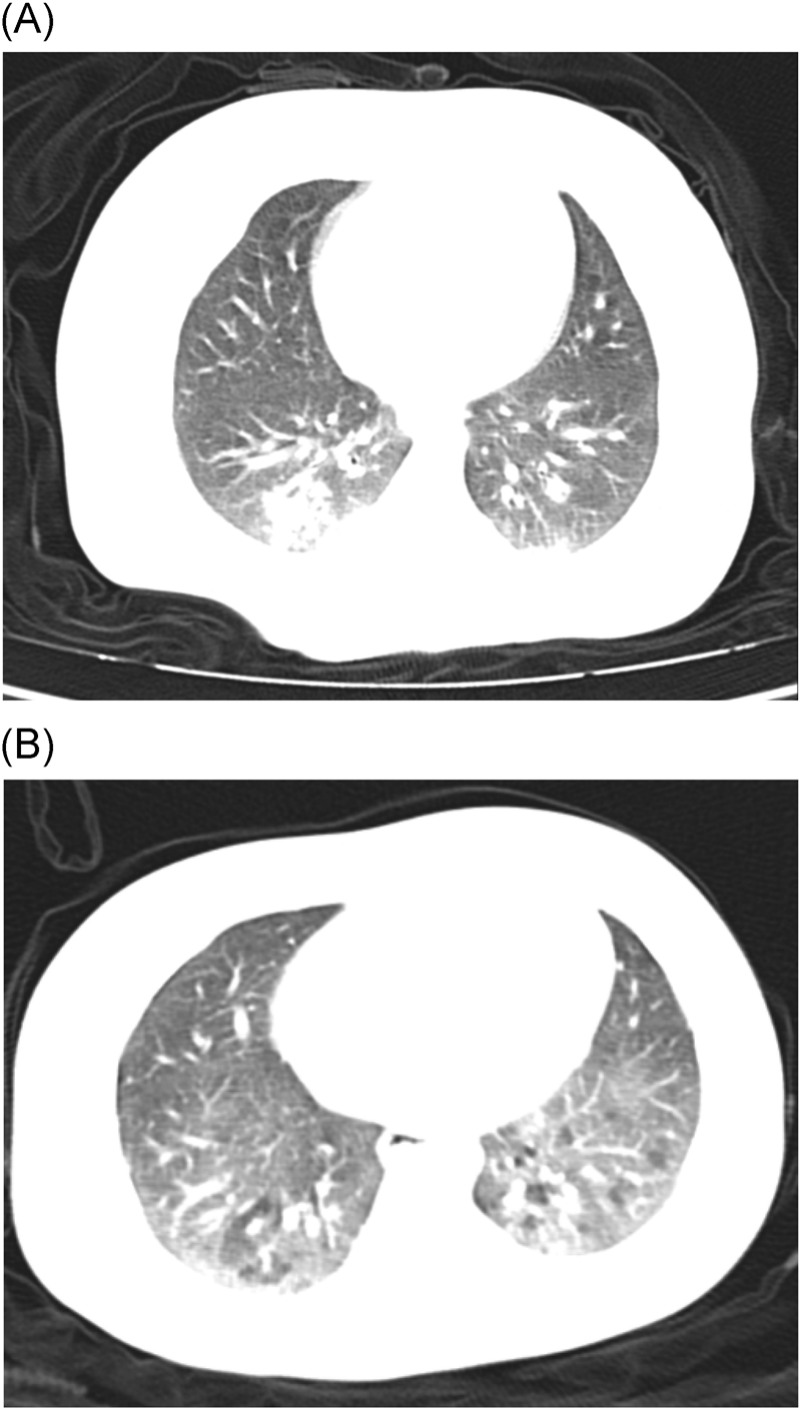
A, The chest computed tomography (CT) of patient 1 showed multiple patchy ground glass opacity shadows in both lungs, and local interlobular septum thickening like a grid. B, The chest CT of patient 2 showed extensive fusion with patchy blurred shadows in both lungs

The patients have been living with their own families in different districts. Patient 1 is the youngest infant diagnosed with Covid‐19 infection in a hospital in Yichang. The summary of their clinical characteristics and treatment is shown in Table 1. Laboratory test results are shown in Table 2. Their chest computed tomographic (CT) images obtained on admission showed bilateral patchy ground glass opacities (Figure 1). Informed consent was obtained from their parents or guardians for the publication of their clinical data.

**Table 1:** Characteristics of two infants hospitalized with COVID‐19 pneumonia

Characteristic	Patient 1	Patient 2
Age, mo	5	7
Sex	Male	Female
Birth weight, kg	3.04	3.35
The general situation at birth	Premature delivery and no asphyxia	Full‐term delivery and no asphyxia
Mode of birth	Cesarean section	Cesarean section
Growth assessment	Average	Above average
Onset time	2020/1/27	2020/2/4
Exposure to source of transmission	Visited Wuhan 11 d before onset	Contacted with a neighbor confirmed with NICP
Characters of other family members	Grandmother was diagnosed with severe NCIP; father and grandfather both have cough	Mother, father, grandmother, and grandfather were all diagnosed with NCIP
Symptoms on admission	Cough, poor appetite (6 d), fever (1 d)	Fever
The highest temperature on admission, °C	37.9	39.5
Mental reaction	Normal	Normal
Coinfections	Not detected	Mycoplasma pneumoniae
Treatment		
Interferon atomization	Yes	Yes
Antivirals	Ribavirin, Abidol	Ribavirin, Abidol, and Oseltamivir
Antibiotics	Amoxicillin clavulanate potassium	Amoxicillin clavulanate potassium and roxithromycin
Intravenous immune globulin	10 g/d (days 1‐3)	10 g/d (day 1)^a^
Glucocorticoids	No	No
Fever duration, d	0	10
Viral duration, d	18	27
Hospitalization, d	15	30

Abbreviations: COVID‐19, coronavirus disease 2019; NCIP, 2019‐nCoV–infected pneumonia.

^a^ Discontinued because of allergic reaction.

**Table 2:** Main laboratory findings on admission of two infants hospitalized with COVID‐19 pneumonia

Laboratory data on admission (reference range^a^)	Patient 1	Patient 2
White blood cell count (3.5‐9.5 × 10^9^/L)	7.92	8.52
Neutrophil count (1.8‐6.3 × 10^9^/L)	0.91	5.49
Lymphocyte count (1.1‐3.2 × 10^9^/L)	6.09^b^	1.84
Platelet count (125‐350 × 10^9^/L)	506	288
Hemoglobin (13.0‐17.5 g/dL)	12.3	10.7
C‐reactive protein (0‐10 mg/L)	2	<0.499
Erythrocyte sedimentation rate (0‐20 mm/h)	23	29
Procalcitonin (<0.05 ng/mL)	<0.05	0.12
Lactate dehydrogenase (120‐250 U/L)	407	274
Creatine kinase (40‐200 U/L)	135	123
Creatine kinase‐MB (0‐25 U/L)	28	25
Alanine aminotransferase (9‐50 U/L)	63	48
Aspartate aminotransferase (15‐40 U/L)	66	62
Total bilirubin (6.5‐8.5 g/dL)	5.6	5.9
Globulin (2.0‐4.0 g/dL)	1.8	1.7
Urea nitrogen (8.0‐22.0 mg/dL^c^)	5.4	9.7
Creatinine (0.64‐1.10 mg/dL^d^)	0.2	0.24
IgG (7‐16 g/L)	5.91	…
IgA (0.7‐4 g/L)	0.35	…
D‐dimer (0‐0.05 mg/L)	0.4	0.35

Abbreviations: COVID‐19, coronavirus disease 2019; IgA, immunoglobulin A; IgG, Immunoglobulin G; MB, muscle and brain type.

^a^ Reference ranges used at Yichang Central People's Hospital for general adults. They may not all be appropriate for children.

^b^ It could be a physiological situation that lymphocyte count of infants before half a year old is significantly higher than the normal value, which may be related to the development of the immune system during this period.

^c^ To convert the values for urea nitrogen to millimoles per liter, multiply by 0.357.

^d^ To convert the values for creatinine to micromoles per liter, multiply by 88.4.

## 3 DISCUSSION

Since 30 January 2020, a total of 47 children (≤12 years of age) with fever, respiratory symptoms, and lung CT suspected viral infection were hospitalized in Yichang Central People's Hospital, of which five were diagnosed with 2019‐nCoV–infected pneumonia (10.6%). All five were from familial clusters. There were at least three to four family members in close contact with the highly suspected or confirmed cases of COVID‐19. So far, all the children have recovered and have been discharged from the hospital.

The two infants presented varying degrees of increase in lactate dehydrogenase and aspartate aminotransferase. We treated them with agents to protect hepatocytes and cardiomyocytes. There is no specific antiviral drug for the treatment of infants with COVID‐19. In our cases, we use three kinds of antivirals for the treatment of COVID‐19 infants for 8 days. And the viral duration of these infants were 18 and 27, respectively. The heart function of infants are impaired, which predict a poor prognosis. One of our cases coinfected with *Mycoplasma pneumonia* has a relative longer duration of symptom release, viral duration, and hospitalization than the other infants who do not. Therefore, the coinfection of COVID‐19 infants should be taken seriously.

The current clinical data show that pregnant women, children, and infants are susceptible to SARS‐CoV‐2. The clinical manifestations of most children are relatively mild. Some children will gradually develop fever, fatigue, dry cough, nasal congestion, runny nose, and other upper respiratory symptoms 3 to 7 days after infection.^2^ Some children just have digestive tract symptoms such as diarrhea, nausea, and vomiting while showing no symptoms of fever or pneumonia.^3^ Most of them recover within 2 weeks and have good prognosis.^3^ But a small number of children may progress to lower respiratory tract infection. Although there was only one death—a 10‐month‐old infant with intussusception died of multiorgan failure—as recorded.^4^ Therefore, the potential risk of death in COVID‐19 infection in infants cannot be ignored, especially when combined with serious or congenital disease.

Also, there are high incidences of influenza, respiratory syncytial virus, and other respiratory infectious diseases in spring. Clinicians are supposed to recognize the possibility of other virus or bacteria, mycoplasma, and other coinfections.

## ETHICS STATEMENT

The study was approved by Medical Ethic Committee of Yichang Central People's Hospital. Informed consent was obtained from their parents or guardians for the publication of their clinical data.

## Data Availability

The data generated within the study is shown in this manuscript. Any raw data or analysis would be available from the corresponding author upon request.
